# Severe Changes in Thymic Microenvironment in a Chronic Experimental Model of Paracoccidioidomycosis

**DOI:** 10.1371/journal.pone.0164745

**Published:** 2016-10-13

**Authors:** Thiago Alves da Costa, Rosária Di Gangi, Rodolfo Thomé, Marina Barreto Felisbino, Amanda Pires Bonfanti, Larissa Lumi Watanabe Ishikawa, Alexandrina Sartori, Eva Burger, Liana Verinaud

**Affiliations:** 1 Department of Structural and Functional Biology, Institute of Biology, University of Campinas (UNICAMP), Campinas, São Paulo, Brazil; 2 Department of Microbiology and Immunology, Institute of Biosciences of Botucatu, Univ. Estadual Paulista (UNESP), Botucatu, São Paulo, Brazil; 3 Department of Microbiology and Immunology, Institute of Biomedical Sciences, Federal University of Alfenas (UNIFAL-MG), Alfenas, Minas Gerais, Brazil; University of Minnesota, UNITED STATES

## Abstract

T cell maturation takes place within the thymus, a primary lymphoid organ that is commonly targeted during infections. Previous studies showed that acute infection with *Paracoccidioides brasiliensis* (Pb), the causative agent of paracoccidioidomycosis (PCM), promotes thymic atrophy that is associated with the presence of yeast cells in the organ. However, as human PCM is a chronic infection, it is imperative to investigate the consequences of Pb infection over the thymic structure and function in chronic infection. In this sense, we developed a new experimental model where Pb yeast cells are injected through the intraperitoneal route and mice are evaluated over 120 days of infection. Thymuses were analyzed in chronically infected mice and we found that the thymus underwent extensive morphological alterations and severe infiltration of *P*. *brasiliensis* yeast cells. Further analyses showed an altered phenotype and function of thymocytes that are commonly found in peripheral mature T lymphocytes. We also observed activation of the NLRP3 inflammasome in the thymus. Our data provide new information on the severe changes observed in the thymic microenvironment in a model of PCM that more closely mimics the human infection.

## Introduction

The thymus is a primary lymphoid organ, responsible for the development of T lymphocytes, major cells in the adaptive immune response. Within the thymus, thymic epithelial cells (TECs) interact with thymocytes, shaping their development throughout different microenvironments of the thymus [1, 2]. Based on their spatial location in the organ and their different roles in thymocyte development, TECs are divided in two distinct subpopulations, medullary TECs (mTEC) and cortical TECs (cTEC) and thus maintenance of the different niches in the thymic architecture is essential for the correct development of the T cells [3, 4]. Previously, the thymus was known as a generative organ isolated from the contact with peripheral cells, being accessible only to progenitor cells [5]. This belief has been long gone, since mounting evidence shows that mature peripheral T cells home to the thymus, recirculate and may even persist in the organ [6]. These mature T cells would be memory T cells that might play a role in keeping the thymus free of foreign antigens thus ensuring the correct development of T lymphocytes [7].

Paracoccidioidomycosis (PCM) is one of the most prevalent systemic mycoses in South and Central America, but also some cases have occurred in non-endemic areas, due to both travelers and immigrant population from these regions [8, 9]. The disease is acquired after inhalation of conidia propagules of *Paracoccidioides brasiliensis* (Pb), a thermally dimorphic fungus that disseminates through lymphatic and circulatory system to the spleen, lymph nodes and skin mostly [10, 11]. This disease is often associated with a depressed cellular immune response that could be associated with production of Th2 cytokines (IL-10, IL-4), reduced function of T cells and failure in antigen presentation [12–17].

We have previously shown that thymic atrophy, in an acute experimental infection model of PCM, is characterized by histological changes, with loss of cortical–medullary delimitation, and the intrathymic presence of parasites [18]. Thymic atrophy has been described as a common consequence of many infectious diseases and metabolic disturbances, such as malaria, Chagas disease, human immunodeficiency virus (HIV) infection, diabetes and malnutrition [19–22]. Recently, Youm *et al*. described a role for the NOD-like receptor family pyrin domain containing 3 inflammasome (NLRP3), in the thymic demise of aged mice. The authors showed enhanced NLRP3 and caspase-1 activity, which leads to IL-1β production that seems to be the major mediator of thymic involution in old age [23]. However, until now these molecules have not been evaluated on a disease-induced-thymic atrophy. The thymic microenvironment integrity must be preserved to ensure the appropriate maturation of T cells since damage to this organ may implicate in inadequate repertoire of T cells in peripheral lymphoid organs, which in turn can lead to autoimmune diseases or immunosuppression. In this context, we have shown that experimental malaria infection leads to thymic atrophy in mice and turns mice susceptible to an aggravated form of experimental autoimmune encephalomyelitis (EAE), the experimental model of multiple sclerosis [24]. More recently, we found that acute infection with Pb yeast cells promotes thymic alterations that lead to a defective repertoire of peripheral T cells [25].

Considering the known tropism of Pb yeast cells for lymphoid organs and a lack of data of experimental chronical infection models, we developed a model of PCM that more closely resembles the human disease. In this model, mice are injected with Pb yeast cells through the intraperitoneal (ip) route and the thymuses are analyzed after 120 days of infection. We found that, although not the natural route of infection, ip injection of yeast cells led to a disseminated fungal infection followed by pathogen invasion in the thymus. Further analyses showed thymic alterations in structure and in cellular subpopulations that are commonly found in secondary lymphoid organs, such as lymph nodes. Our data suggest that, in chronic PCM, the thymus undergoes morpho-functional changes ultimately acquiring characteristics of secondary lymphoid organ.

## Materials and Methods

### Animals

*Specific pathogen-free* BALB/c male mice with 6–8 week-old, were purchased from Centro Multi-Institucional de Bioterismo (CEMIB) and maintained in transparent acrylic plastic isolators in ventilated racks (Alesco, SP, Brazil) on a 12h light/dark cycle and controlled temperature environment (20°-24°C) throughout the study, with sterile water and food (Nuvilab CR-1; Nuvilab, PR, Brazil) provided *ad libitum*. Once a week, the bedding of the isolators was changed with autoclaved wood shavings, and the mice were accompanied to assess their health. The animals showed no signs of severely illness, as they maintained their common behavior regarding food consumption and movement. No animals died prior to our experimental endpoint. This study was conducted according to the ethical principles of animal research adopted by the Brazilian National Council for the Control of Animal Experimentation (CONCEA) and was approved and carried out in accordance with the guidelines of the Institutional Committee for Use of Laboratory Animals (CEUA/UNICAMP protocol number # 2968–1).

### Fungus and infection

Virulent isolate of *P*. *brasiliensis*, named Pb18, kindly provided by Prof. Vera L. G. Calich (University of São Paulo/Brazil), was used in this study. Yeast cells were grown at 37°C in Fava-Netto’s medium and used at the seventh day of growth, as previously described [26]. The fungal mass was suspended in phosphate-buffered saline (PBS, 0.02 M pH 7.2), mixed twice for 10 seconds on a Vortex-mixer, centrifuged and double washed in PBS. The concentration was adjusted to 1x10^7^ yeasts/mL based on hemocytometer counts. Viability was determined by Trypan blue staining [27] and was higher than 90%. Mice were injected intraperitoneally with 5x10^6^ yeast/animal contained in 0.5 mL of PBS or with PBS alone. Opposed to the intra-tracheal infection where the lungs are the primary focus, intraperitoneal route of infection was chosen due to a chronical aspect of infection and evenly dissemination of the fungal burden to internal organs similar to severe cases of human chronical PCM.

Infection was carried out for one hundred and twenty days when mice were euthanized by deep anesthesia with ketamine/xylazine (100 mg/kg ketamine hydrochloride and 5 mg/kg xylazine hydrochloride), and the thymuses were excised. Groups of five mice were used for each experiment.

### Histological analysis

Thymuses from infected and control mice were collected, weighted and fixed individually in 4% paraformaldehyde solution for 16 hours at 4°C. The specimens were submitted to diafanization with xylene, dehydrated by graded ethanol, embedded in paraffin and cut in 5-μm-thick sections. Histopathological analyses were evaluated on sections stained with hematoxylin and eosin (H&E), and to highlight fungus invasion, sections were impregnated with silver in accordance with Grocott’s methenamine silver staining (GMS) [28, 29].

### Flow cytometry

Thymuses were homogenized individually in staining buffer (PBS 0.02 M pH 7.2 enriched with 2% Fetal Calf Serum) and cell number was estimated by hemocytometer. Multi-color flow cytometric phenotypic analysis, cell death assessment and intracellular detection of cytokines were performed with a Gallios Flow Cytometer (Beckman Coulter, CA, USA) using directly conjugated anti-mouse monoclonal antibodies: anti-CD3/PE-Cy7 (clone 145-2C11), anti-CD4/FITC (clone H129.19), anti-CD4/PECy5 (clone RM4-5), anti-CD44/PE (clone IM7), anti-MHC-II/PERCP-Cy5.5 (M5/114.15.2) from (BD Pharmigen, CA, USA), anti-CD8/APC (clone 53–6.7), anti-CD24/FITC (clone M1/69), anti-CD45/PE-Cy7 (clone 30-F11), anti-Ly51/FITC (clone FG35.4), IFN-γ/PE (clone XMG1.2) and IL-17/FITC (clone eBIO17B7) (eBioscience, CA, USA). For cell death assessment, annexin-V and propidium iodide staining were performed according to the manufacturer’s instructions (BD Pharmigen, San Jose, CA, USA). For intracellular detection of cytokines, thymic cells were stimulated with phorbol 12-myristate 13-acetate (PMA, 50 ng/mL, Sigma-Aldrich, MO, USA) and ionomycin (500 ng/mL, Sigma-Aldrich) in the presence of brefeldin A (1 μg/mL, Sigma-Aldrich) for 4h at 37°C. Later, cells were surface stained with anti-CD4/PECy5 (clone RM4-5) and anti-CD8/APC (clone 53–6.7) for 20 minutes at 4°C, fixed and permeabilized with Fixation/Permeabilization Buffer (eBioscience, CA, USA). Antibodies against IFN-γ/PE. (clone XMG1.2) and IL-17/FITC (clone eBIO17B7) were added and the suspensions were incubated at 4°C for 30 minutes. Isotype controls were used as well. Analyses were performed after recording 20.000 events for each sample and data analyzed using FlowJo vX.0.7 (Tree Star Inc., Ashland, OR, USA).

### Gene expression

Relative levels of mRNA from genes of cytokines and caspases were evaluated by real-time quantitative polymerase chain reaction (qPCR). Thymus RNA was extracted individually, converted to cDNA using commercial kits (High Capacity cDNA Reverse Transcription Kit, Applied Biosystems, Foster City, CA) and RT-PCR reactions were carried out with TaqMan PCR Master Mix (Applied Biosystems, Foster City, CA), according to manufacturer’s recommendations, using different sets of oligonucleotides and probes for amplification of the following mRNA: Glyceraldehyde 3-phosphate dehydrogenase GAPDH (endogenous control), interleukin (IL)-1β, IL-4, IL-6, IL-17, IL-18, IFN-γ, TNF-α, transforming growth factor-beta (TGF-β), caspase-1, caspase-3, caspase-8 and NLRP3 genes. These corresponded respectively to the following reference numbers (Applied Biosystems): Mm99999915_g1, Mm00434228_m1, Mm00445259_m1, Mm00446190_m1, Mm00439619_m1, Mm00434226_m1, Mm00801778_m1, Mm00443258_m1, Mm01178820_m1, Mm00438023_m1, Mm01195085_m1, Mm00802247_m1 and Mm00840904_m1. Data are presented as relative mRNA levels calculated by using the method of delta threshold (2^-**ΔΔ**Ct^). **Δ**Ct (**Δ**Ct = Ct of target gene minus Ct of GAPDH).

### Protein quantification

Total protein was extracted from thymus of control and infected mice using RIPA lysis buffer, following manufacturer’s instructions (Millipore, CA, USA). Protein concentration was measured by the Bradford method, following manufacturer’s instructions (Bio-Rad, CA, USA). Cytokines were detected by Enzyme-Linked Immunosorbent Assay (ELISA) using commercial kits (R&D Systems, MN, USA). IL-1β, IL-17, IL-18, and IFN-γ were detected in protein extracts.

#### Immunobloting

To analyze NLRP3 inflammasome activity we performed immunobloting for NLRP3 and caspase-1. After protein concentration measurements, equal amounts of samples were separated in a 8% or 12% sodium dodecyl sulfate polyacrylamide gel electrophoresis (SDS-PAGE) as described by Laemmli [30].

The immunobloting technique was performed as described by Towbin *et al* [31]. The membrane was incubated overnight with a rabbit anti-caspase-1 (sc514; Santa Cruz Biotechnology, Inc., TX, USA) or a goat anti-NLRP3 antibody (ab4207, Abcam, CA, UK) in a blocking solution. After washing, secondary antibody anti-rabbit HRP or anti-goat HRP was added (sc2004; sc2020; Santa Cruz Biotechnology, Inc., TX, USA). Immunoreactive proteins were determined using an ECL Western blotting detection system (Amersham, PA, USA). GAPDH (sc25778 HRP; Santa Cruz Biotechnology, Inc., TX, USA) was used as the loading control for both NLRP3 and caspase-1. Image J software (NIH, MD, USA) was used for estimation of pro-caspase-1, the active form of caspase-1, and NLRP3 inflammasome assembly, through a GAPDH ratio.

### Statistical analysis

Student’s *t*-test was used for statistical evaluation of results comparing infected and control animals. Results are expressed as mean values ± Standard Error of the Mean (SEM), and p values lower than 0.05 were considered statistically significant.

## Results

### Chronic infection with *Paracoccidioides brasiliensis* provokes thymic atrophy and organ invasion by yeast cells

**Fig 1:**
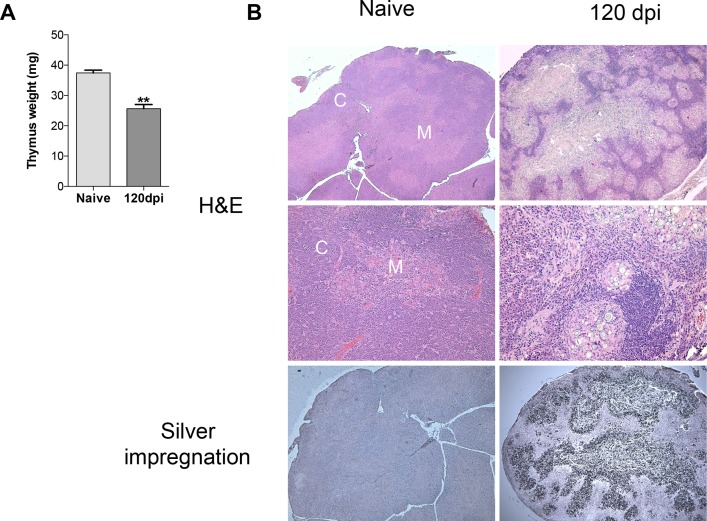
Prolonged *Paracoccidioides brasiliensis* infection leads to thymic atrophy and intense fungal burden. Male BALB/c mice (n = 5 mice/group for each analyses per experiment replicate) were inoculated with 5x10^6^ Pb18 yeasts contained in PBS or with only PBS (control group), intraperitoneally. One hundred and twenty days after inoculation, mice were killed and the thymus removed for analysis. A) Thymus from the 120dpi group was smaller in size and weighted less than the naive group. (B) Hematoxilin-Eosin staining showed histologic disorganization in 120dpi, and no evidence of typical cortical (C) and medullary (M) regions, while naive mice showed normal histologic distribution. In more detail below, giant cells and granuloma formation is present in 120dpi. Silver impregnation revealed massive fungal infiltration in the thymic medulla in 120dpi. Statistical analysis was carried out with Student’s t-test. **p<0.01. Results are expressed by Mean ± SEM. Images are representative of three independent experiments with similar results. The images were taken in an Olympus BX50. Magnification 40x (upper and lower panels); 100x (middle panel).

Our group recently investigated the effects of acute infection of *Paracoccidioides brasiliensis*. We found that after seven days of infection, the thymus of BALB/c mice remained mostly unchanged regarding the delimitation of cortex and medulla (*data not shown [25]*). However, we found that after 120 days of infection, thymuses from infected mice underwent profound weight loss compared to healthy mice (Fig 1A). Hematoxilin-Eosin (H&E) staining of thymic sections revealed a completed disorganized stromal cell compartment where cortical and medullary histologic regions could not be recognized (Fig 1B, right upper panel). Giant cells and granuloma formation were also presented in thymus of chronically infected mice, but not in thymus from healthy mice (Fig 1B, right middle panel). Interestingly, giant cells and granuloma were surrounding viable yeast cells, found in massive amounts in the medulla (Fig 1B, right lower panel).

### Decreased cellularity of thymocytes and thymic epithelial cells followed by high apoptosis index in chronically infected mice

**Fig 2:**
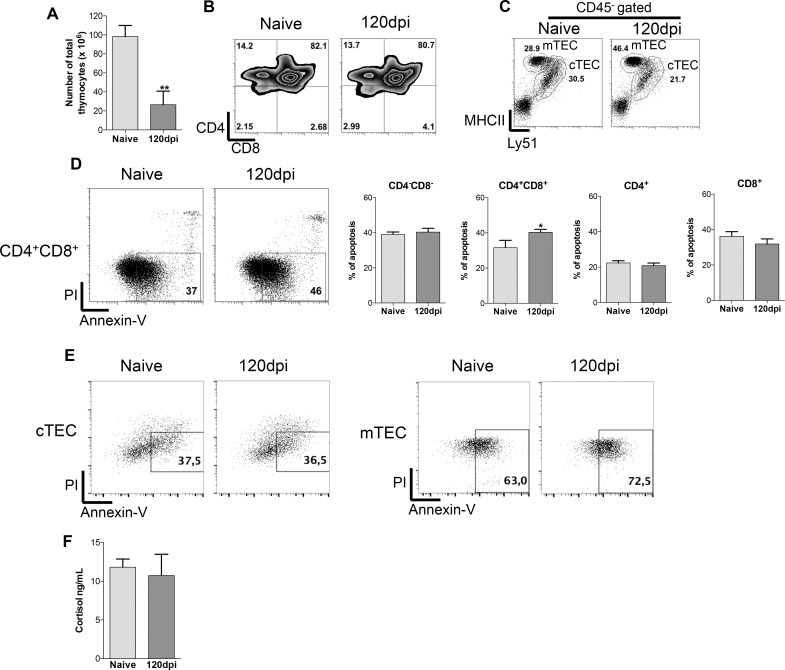
*Paracoccidioides brasiliensis* infection leads to decreased thymocytes and TECs cellularity with increased cell death of CD4^+^CD8^+^ thymocytes and mTEC without cortisol contribution. Male BALB/c mice (n = 5 mice/group for each analyses per experiment replicate) were inoculated with 5x10^6^ Pb18 yeasts contained in PBS intraperitoneally or with only PBS as control group. One hundred and twenty days after inoculation mice were killed, the thymus and the blood were collected and processed individually for analysis. A) Total thymocytes numbers were lower in 120dpi compared to the naive group. B) Frequency of thymocytes subpopulations remained unchanged between naive and 120dpi groups. C) Frequency of thymic epithelial cells (TEC) subpopulations changed considerably in 120dpi, higher percentages of mTEC were found while cTEC frequency decreased in comparison to the naive group. D) Higher percentage of apoptotic double positive thymocytes from the 120dpi group compared to the naive group, but not in other thymocytes subpopulations. E) Higher percentage of apoptotic medullary thymic epithelial cells (mTEC) from the 120dpi group compared to the naive group, while no alterations in cTEC apoptosis was found. F) Cortisol production was not altered between the naive and 120dpi groups. At least 20000 events were analyzed with FlowJo vX.0.7 (Tree Star Inc., Ashland, OR, USA). Statistical analysis was carried out with Student’s t-test. *p<0.05; **p<0.01; ***p<0.001. Representative data from three independent experiments with similar results.

Given that after 120 days of infection the thymus is rendered atrophic and harbors yeast cells, we aimed to evaluate the cellular subpopulations in the thymus. Firstly, we evaluated the absolute cell numbers of thymocytes and TEC subpopulations frequency. The absolute numbers of thymocytes, in infected mice, were drastically reduced when compared to the control group (Fig 2A). We observed no differences in thymocytes frequencies between the control and infected group (Fig 2B). The frequencies of TECs subpopulations changed considerably. Frequency of mTECs from infected mice doubled while cTECs was reduced compared to cells from naive mice (Fig 2C).

Next, we evaluated if cell death played a role altering the cell frequencies. We observed that samples from infected mice presented a higher percentage of thymocytes expressing the apoptotic marker (annexin-V) in CD4^+^CD8^+^ (double positive—DP) compartment but not in other subpopulations compared to healthy mice (Fig 2D). Interestingly, although mTEC frequency increased in infected mice, these cells also presented higher apoptotic frequency compared to cells from healthy mice (Fig 2E). Apoptotic ratio of cTECs remained unchanged between groups (Fig 2E). One could imagine that higher apoptotic frequencies of thymocytes and mTECs is linked to cortisol levels. We found that serum cortisol levels were similar between healthy and infected mice (Fig 2F), which excludes the hypothesis that cortisol is driving apoptosis of cells in infected mice.

### Increased production of inflammatory cytokines, inflammasome activity and initiator caspase-8 gene expression in mice infected with *Paracoccidioides brasiliensis*

**Fig 3:**
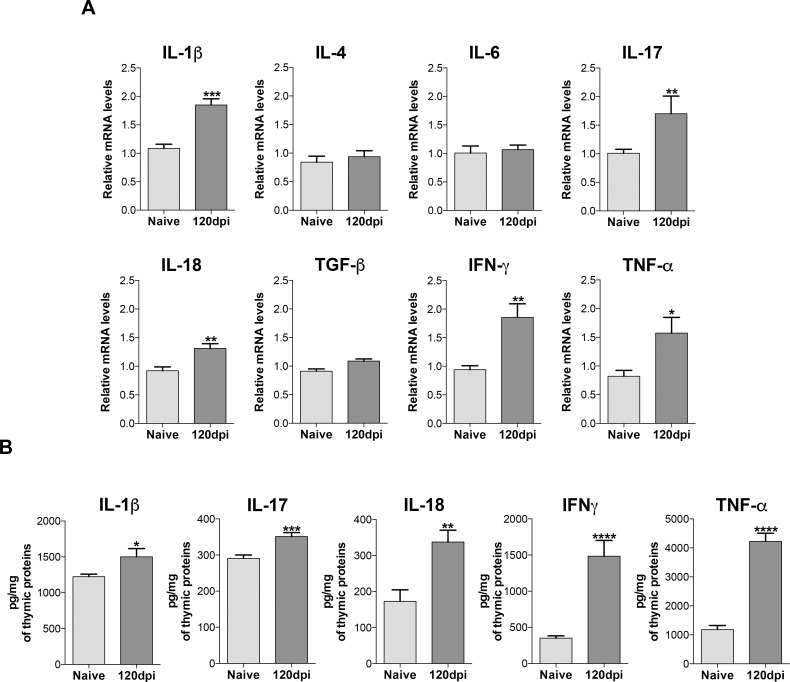
Increased inflammatory cytokines gene expression and protein levels in the thymus of infected mice. Male BALB/c mice (n = 5 mice/group for each analyses per experiment replicate) were inoculated with 5x10^6^ Pb18 yeasts contained in PBS intraperitoneally or with only PBS (control group). One hundred and twenty days after inoculation, mice were killed and the thymus was collected and processed individually for analysis. A) Increased gene expression of IL-1β, IL-17, IL-18, IFN-γ and TNF-α from 120dpi group compared to the naive group. B) Protein levels of the inflammatory cytokines also showed significant increase in 120dpi group compared to the naive group. Statistical analysis was carried out with Student’s t test. *p<0.05; **p<0.01; ***p<0.001; ****p<0.0001. Representative data from three independent experiments with similar results. Expression levels of genes were represented as a relative copy numbers by using the method of delta threshold (2^-ΔΔCt^).

Since cortisol remained unchanged during infection, we aimed to investigate which signals could lead to increased apoptosis in cells from infected mice. We found an increased gene expression of the pro-inflammatory cytokines IL-1β, IL-17, IL-18, IFN-γ and TNF-α in thymus from infected mice compared to naive mice (Fig 3A). No significant alterations were observed in expression of IL-4, IL-6 and TGF-β (Fig 3A). Protein dosage of these cytokines followed the gene expression pattern, with higher levels of IL-1β, IL-17, IL-18, IFN-γ and apoptosis-inducing TNF-α in thymuses from infected mice in contrast to samples from healthy mice (Fig 3B).

**Fig 4:**
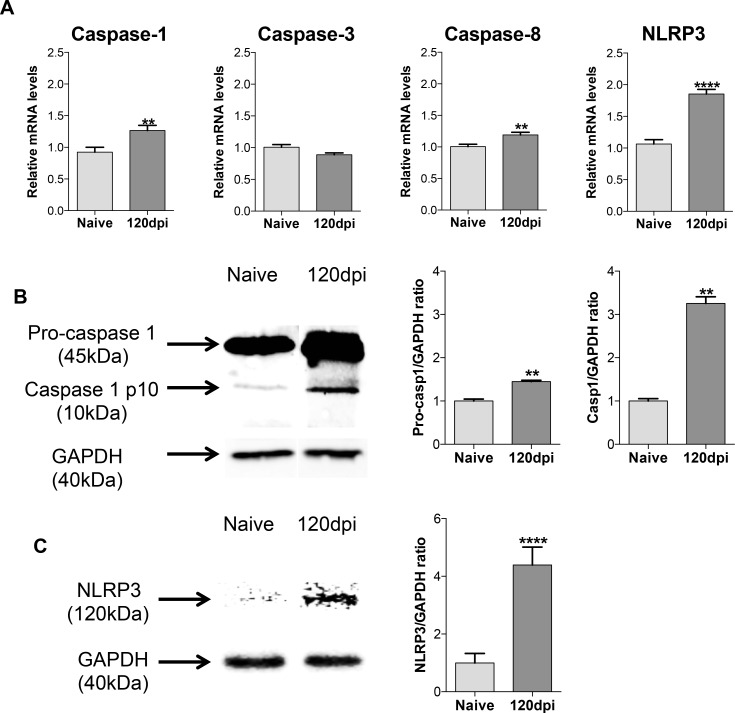
Increased inflammasome and caspase-1 activity in the thymus of infected mice. Male BALB/c mice (n = 5 mice/group for each analyses per experiment replicate) were inoculated with 5x10^6^ Pb18 yeasts contained in PBS intraperitoneally or with only PBS (control group). One hundred and twenty days after inoculation, mice were killed and the thymus was collected and processed individually for analysis. A) Increased initiator caspase-8 gene expression on 120dpi group compared to the naive group. Increased inflammatory caspase-1 gene expression on 120dpi group compared to the naive group. Increased NLRP3 inflammasome gene expression on 120dpi group compared to the naive group. B) Increased pro-caspase-1 production and increased caspase-1 activity on 120dpi group compared to the naive group. C) Increased NLRP3 inflammasome complex assembly on 120dpi compared to the naive group. Statistical analysis was carried out with Student’s t-test. **p<0.01, ****p<0.0001. Representative data from three independent experiments with similar results. Expression levels of genes were represented as a relative copy numbers by using the method of delta threshold (2^-ΔΔCt^). Image J software (NIH, MD, USA) was used for the estimation of the pro-caspase-1, the active form of caspase-1 and NLRP3 inflammasome assembly, through a GAPDH ratio.

We also aimed to investigate the gene expression levels of caspases and inflammasome activity. Caspases are enzymes involved in many aspects of the immune response, from cleavage of pro-IL-1β to its active form (by caspase-1) to induction of apoptosis (initiator caspase-8 and executioner caspase-3) [32]. We found that, although no significant alterations were found in the expression of caspase 3, thymus from infected mice presented higher expression of “inflammatory” caspase 1 and initiator caspase-8 compared to thymus from healthy mice (Fig 4A). As caspase-1 gene expression was higher in infected thymuses, we also investigated if NLRP3 inflammasome, as seen by Youm *et al* in aged mice, would have a role on the increased cytokine production [23]. The NLRP3 gene expression increased on the thymus of infected mice (Fig 4A), and by immunobloting quantification both caspase-1 activity and NLRP3 assembly were enhanced on those organs (Fig 4B and 4C, respectively). These data suggest that in chronically infected mice, the thymus undergoes significant changes in cellular constitution and produces inflammation-linked mediators. These changes could lead to a defective function of the thymus in *Paracoccidioides brasiliensis* infection.

### Recirculating mature T cells home back to infected thymuses, leading to increased frequency of cytokine producing Th17 and T CD8^+^ IFN-γ

**Fig 5:**
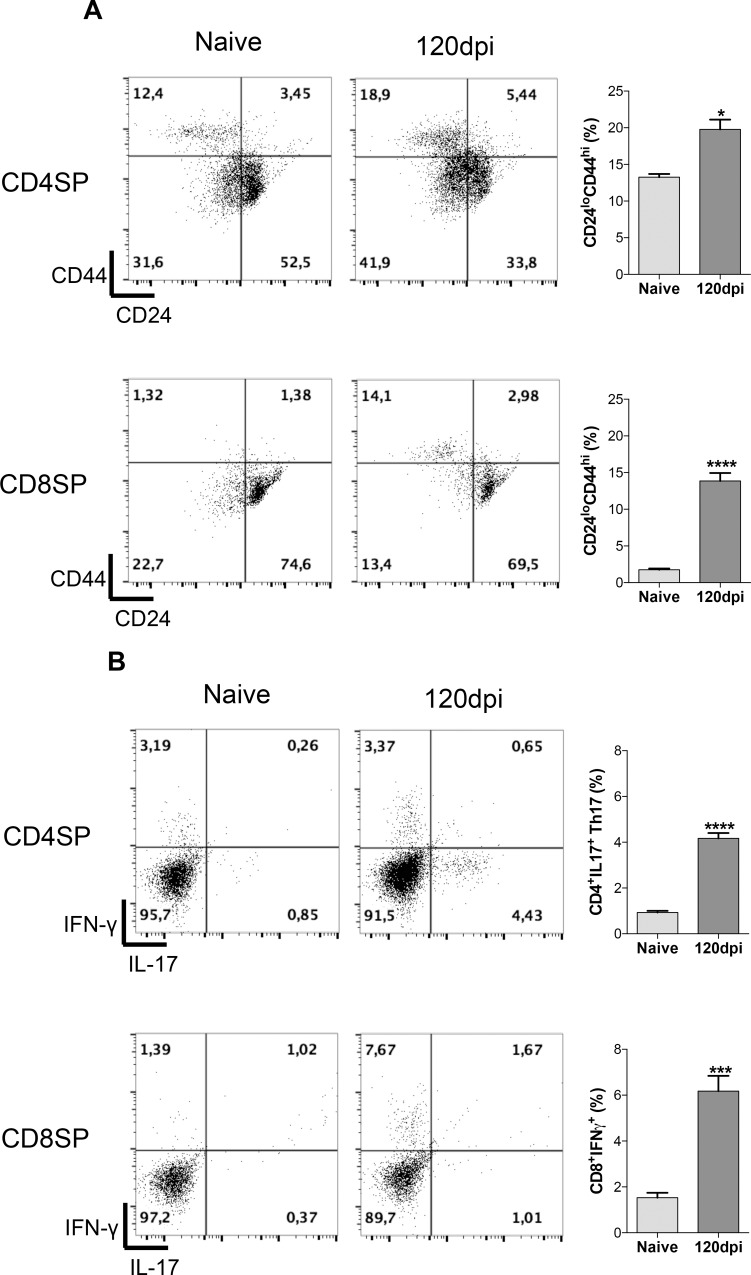
Recirculating mature T cells home to infected thymuses, leading to increased frequency of cytokine producing Th17 and T CD8^+^ IFN-γ^+^. Male BALB/c mice (n = 5 mice/group for each analyses per experiment replicate) were inoculated with 5x10^6^ Pb18 yeasts contained in PBS intraperitoneally or with only PBS (control group). One hundred and twenty days after inoculation, mice were killed and the thymus was collected and processed individually for analysis. A) Frequency of CD44^hi^CD24^lo^ T cells increased among CD4^+^ and CD8^+^ subpopulations in 120dpi compared to the naive group. B) Cytokine producing T cells, Th17 and CD8^+^IFNγ^+^ was found in 120dpi, while practically absent in the naive group. Representative data from three independent experiments with similar results. At least 20000 events were analyzed with FlowJo vX.0.7 (Tree Star Inc., Ashland, OR, USA). Statistical analysis was carried out with Student’s t-test. ***p<0.001, ****p<0.0001. Representative data from three independent experiments with similar results.

After acknowledging that infected thymuses showed increased production of inflammatory cytokines, we wondered if such observation would be attributed to mature T cells that re-enter the thymus in an attempt to eliminate the pathogen.We found a higher frequency of CD44^hi^CD24^lo^ mature T cells from both subpopulations, CD4 single-positive (SP) and CD8SP, within the thymus of infected mice (Fig 5A). CD8^+^ T cells showed the highest increase in frequency of CD44^hi^CD24^lo^ markers. These are likely peripheral cells that were peripherally activated and returned to the thymus [7, 33].

To further link the increased inflammatory profile of infected thymuses with the recirculating T cells, we proceeded to evaluate intracellular detection of cytokines (IL-17 and IFN-γ) within thymic subpopulations of T cells. We detected higher frequencies of Th17 (CD4^+^ IL-17 producing cells) and CD8^+^ IFN-γ producing T cells within the thymus of infected mice (Fig 5B).

## Discussion

In this paper, we show that the thymus from mice chronically infected with *Paracoccidioides brasiliensis* presented a severe involution, disarranged cell constitution, granuloma formation and production of inflammatory mediators that cumulatively resembles a secondary lymphoid organ. These data are intriguing and, to our knowledge, we are the first group to characterize the consequences in the thymic architecture during a prolonged *P*. *brasiliensis* infection.

The thymic microenvironment is supposed to be safeguarded from circulatory inflammatory cells ensuring correct T cell development, due to the presence of the blood-thymus barrier that configures the thymus as an immune privileged organ [5]. However, blood-thymus barrier is limited to the cortex region and the constant influx of bone marrow precursors and macrophages through the cortico-medullary region makes the thymus only partially isolated from the blood stream [5, 34]. Over the past years, several authors have shown that pathogens are able to invade the thymus, “breaking” its immune-privileged status. Despite being a target organ of many infectious diseases that lead to organ atrophy, the presence of inflammatory cells infiltration is rarely seen in the thymus [35]. In a mycobacterial infection model, thymus is colonized by *Mycobacterium spp*. and, despite the increased bacterial load, no granulomas were found in the organ [36]. Sotomayor and colleagues made different observations on *Cryptococcus neoformans* infected rats, although present on the thymus, the pathogen leads to increased thymic weight characterized by cortical hyperplasia with slight granulomatous reaction and germinal center formation [37]. Regarding this fact, our findings show that thymic alterations in prolonged Pb infection lead to a rupture in the immune privileged status, with infiltrating inflammatory cells forming granulomas in response to the massive yeast invasion.

A common feature in acute-infection models is the severe atrophy of the thymus, mainly due to apoptosis related depletion of CD4^+^CD8^+^ thymocytes, the major cellular type in the organ [35, 38, 39]. Notwithstanding, CD4^+^CD8^+^ thymocytes absolute numbers were very reduced in infected mice, and remaining cells showed a higher rate of apoptosis in comparison to the non-infected mice. Probably the reduction in other thymocytes subpopulations was due to higher apoptosis frequencies throughout the infection and, after 120 days of inoculation, these thymocytes subpopulations in infected mice are at much lower numbers than the control group, and therefore the thymic selection itself leads to a higher apoptosis index than the infection.

The imbalance between different thymic and splenic subpopulations can also affect the peripheral immune response as seen in *Cryptococcus neoformans* infected rats, where the authors observed significant increase on the cells expressing the class II MHC IE molecule on the thymus and spleen, linking them to the down regulation of the immune response seen in this infection model [37, 40].

A higher production of pro inflammatory cytokines, as observed by increased gene expression and protein quantification, causing a stressful environment for thymic cells may explain the fewer numbers of thymocytes and thymic epithelial cells found in the infected group, since those cytokines are known to directly contribute to the thymocytes depletion in infections [41, 42]. In addition, increased expression of caspase 1 and caspase 8 may account for higher apoptosis index found in these animals, through inflammation and direct cell death. According to some authors, caspase-8, thymocytes depletion and cortisol production are intertwined [43, 44]. In our infection model, as seen in an influenza induced thymic atrophy [42], cortisol production has no role in the thymic atrophy.

We believe that augmentation of intrathymic production of inflammatory cytokines, such as IFN-γ, IL-17 and TNF-α may be related to the participation of effector T cells which home to the thymus to control infection. Indeed, we observed an increased frequency of CD44^hi^CD24^lo^ T cells in thymus of infected mice. This phenotype may correspond to recirculating T cells that reenter the thymus, as seen by others [7, 33, 45]. In addition, presence of Th17 and CD8^+^ IFN-γ producing cells in infected thymus indicates that a pro-inflammatory environment is maintained by mature T cells that likely respond to *Paracoccidioides brasiliensis*. Nobrega and colleagues made a similar observation in a model of *Mycobacterium sp*. infection [46].

We also investigated the participation of NLRP3 inflammasome, which is a multiprotein cytoplasmic complex, since we noted increased production of IL-1β and IL-18. NLRP3 can be activated by a wide range of stimuli including endogenous danger signals and microbial products [47, 48]. Recently, NLRP3 inflammasome activation has been detected *in vitro* on bone-marrow dendritic cells upon *Paracoccidioides brasiliensis* challenge, showing an important role for pathogen clearance [49]. In our model, NLRP3 protein expression and caspase-1 activity were increased in thymus from infected mice, which suggest that there might be a response to the pathogen invasion locally, in an attempt to reduce fungal burden. It seems that production of IL-1β is linked to thymic macrophages or dendritic cells, and the intrathymic production of such cytokine would impact the TEC compartment, as is also reported by other authors [23, 50]

Altogether, we show that the thymus of chronically infected mice is severely compromised. Our data suggest that thymus from *Paracoccidioides brasiliensis*-infected mice may no longer function as a primary organ of the immune system but rather exert activities related to secondary lymphoid organs. Further studies are needed to evaluate the role of the thymus during fungal infection and whether invasion of the organ by yeast cells could be associated with peripheral immunosuppression frequently observed during *Paracoccidioides brasiliensis* disease.
