# Correction: Severe Changes in Thymic Microenvironment in a Chronic Experimental Model of Paracoccidioidomycosis

**DOI:** 10.1371/journal.pone.0168810

**Published:** 2016-12-16

**Authors:** Thiago Alves da Costa, Rosária Di Gangi, Rodolfo Thomé, Marina Barreto Felisbino, Amanda Pires Bonfanti, Larissa Lumi Watanabe Ishikawa, Alexandrina Sartori, Eva Burger, Liana Verinaud

The citation appears incorrectly in the published article. The correct citation is: Alves da Costa T, Di Gangi R, Thomé R, Felisbino MB, Bonfanti AP, Ishikawa LLW, et al. (2016) Severe Changes in Thymic Microenvironment in a Chronic Experimental Model of Paracoccidioidomycosis. PLoS ONE 11(10): e0164745. doi:10.1371/journal.pone.0164745
